# Computer modeling of digestive processes in the alimentary tract and their physiological regulation mechanisms: closing the gap between digestion models and *in vivo* behavior

**DOI:** 10.3389/fnut.2024.1339711

**Published:** 2024-03-28

**Authors:** George A. van Aken

**Affiliations:** Insight Food Inside, Breda, Netherlands

**Keywords:** digestion, digestive system, gastro-intestinal, alimentary system, digestion modeling, *in silico* digestion modeling, organ-on-a-chip, pharmaceutical modeling

## Abstract

**Introduction:**

A model has been developed for in silico simulation of digestion and its physiological feedback mechanisms.

**Methods:**

The model is based on known physiology described in the literature and is able to describe the complexity of many simultaneous processes related to food digestion.

**Results:**

Despite the early stage of development of the model, it already encompasses a large number of processes that occur simultaneously, enabling the prediction of a large number of post-prandial physiological markers, which can be highly functional in combination with in vitro, organ-on-a-chip and digital twin models purposed to measure the physiological properties of organs and to predict the effect of adjusted food composition in normal and diseased states.

**Discussion:**

Input from and collaboration between science fileds is needed to further develop and refine the model and to connect with *in vitro*, *in vivo*, and *ex vivo* (organ-on-a-chip) models.

## Introduction

The healthy life of humans and animals requires efficient processes of digestion and absorption of food by the alimentary system. To this end, the alimentary system breaks down the food into small molecular species by diminution of food material by mastication and gastric grinding and by enzymatic digestion into molecular species that can be absorbed by the gastrointestinal tract and unabsorbed residues that are fermented by gut microbes. These processes are complex, strongly dependent on the food material, and also vary among individuals (sex, age, and health conditions). To control and optimize the uptake of orally administered nutrients or pharmaceutical ingredients and to keep healthy gastrointestinal conditions, many studies have been conducted to identify and model these digestion and absorption processes.

Focusing on food digestion in humans, much understanding of the mechanical, enzymatic, and fermentative breakdown of food materials has been obtained from *in vivo* studies, analyzing the behavior of separate digestive organs (mouth, stomach, small intestine, and large intestine), by tracking the motility, digestive fluid release, and progression of digestion through imaging and sampling techniques (1–12) in healthy and diseased states and from studies on animals. These studies have been supplemented by a large number of *in vitro* and *ex vivo* digestion studies, using laboratory setups in which enzymes, microbes, or simulated secretions from digestive glands have been added to food material (13, 14), and cell biological studies (15–17). Emerging are the *organ-on-a-chip* models, where the physiology of gastrointestinal tissues and whole organs can be studied in detail (18, 19).

For pharmaceutical applications, pharmacokinetic/pharmacodynamic (PK/PD) computer (“*in silico*”) models are available to model dose–concentration–response relationships and describe and predict the time-dependent effects of a drug dose (20) by calculating the bioavailability of the pharmaceuticals, taking into account processes such as the dissolution of pharmaceuticals with defined particle size and applying advanced models of absorption of the dissolved pharmaceuticals and their post-absorptive effects. Current models provide realistic descriptions of absorption, distribution over the body, metabolism by various organs and the excretion from the body (21), and recently are being improved for describing the effect of personal differences by implementing statistical tools to directly include clinical observations into the predictive models (22). Examples of the active PKPD platforms are Simcyp 13.1 (23), GastroPlus 8.0 (24), and GI-Sim 4.1 (25).

Although such pharmaceutical models are highly functional for modeling post-absorptive processes in body tissues for pharmaceutical purposes, they are not well-equipped for a detailed modeling of food digestion. For this reason, they could benefit from an improved modeling of the pre-absorptive processing in the alimentary tract in a fed state (20, 26, 27). In the current pharmaceutical models, the GI tract is usually kept relatively simple, for example, describing the stomach by a single mixed compartment with a fixed or pre-programmed time-dependent pH, the description of gastric emptying rate at best limited by the viscosity of the gastric content and caloric output, and the small intestine as a line-up of a few well-mixed compartments with similar properties and constant rates of transport between these compartments. The presence of food is typically described by distinguishing fed and non-fed states only, without taking the precise composition and digestive properties of the food into account.

The first steps have been undertaken to couple *in vitro* digestion modeling by the TIM-1 gastrointestinal model (28, 29) to PK/PD computer modeling (30) in order to make the *in vitro* modeling conditions more dynamic, recognizing that many physiological conditions vary in time and adjust to the content of the gastrointestinal compartments. In a recent development, the *in vitro* part of the modeling has also been incorporated in a digitized form into a GastroPlus-based modeling program (31).

The computer program discussed in this publication is a mechanistic digestion model (MDM) that has been developed over the last decade by the author, intending to describe the digestion of food and the development of foods with targeted digestion behavior. It models in detail the processes of digestion and the physiology of a large number of hard-wired physiological processes and their interactions involved in nutrition, including the desire to eat and regulation of food intake (involving sensations of hunger, fullness, and satiety), oral processing and the swallowing reflex, gastro-intestinal processing, gastro-intestinal transit, transfer through the mucus border to the absorptive tissue, absorption, some post-absorptive processes, and the physiological regulation mechanism that are based on the signals of receptors all along the mouth and gastrointestinal tract, with the physiological purpose to control consumption, optimize nutrient absorption, and reduce the potential harm of the digestive enzymes to the gastrointestinal tissues.

The MDM attempts to combine the large amount of available mechanistic knowledge based on a large number of studies (*in vivo*, *in vitro*, and *ex vivo*) available from the literature into a predictive model (32). The MDM currently describes digestive processes for the averaged healthy human condition, for which some of the parameterizations of the conditions (such as the concentration of digestive enzymes) during digestion have been aligned with the INFOGEST static *in vitro* simulation protocol of gastrointestinal food digestion (13) but can also be used to describe and model altered physiology, such as the effects of age, diseases, and surgical interventions, by adapting the model parameters. The current model has been used to quickly assess the expected effect of altered food structures, food compositions, and changes in physiological states and to get insight into the complexity of digestion. It is being used to support food development and can support the further development of PKPD and digital twin models for the human body. An advantage of a mechanistic model is that it allows extrapolation of *in vivo* outcomes toward different meal compositions, consumption rates, and physiological conditions in humans and is extendable to different physiologies of (mammal) species. In the context of *organ-on-a-chip* models, the MDM may enable to closing the gap between artificial intestine and organ-on-a-chip models and allowing the prediction of *in vivo* outcomes. The MDM may help to define the appropriate conditions for an *organ-on-a-chip* model, for example, in the fed state. In return, the insights gained from *organ-on-a-chip* models can be modeled and then used to improve the modeling of organs in the MDM. This study gives a concise description of the MDM in its current form and demonstrates its predictive value with some examples.

**Figure 1:**
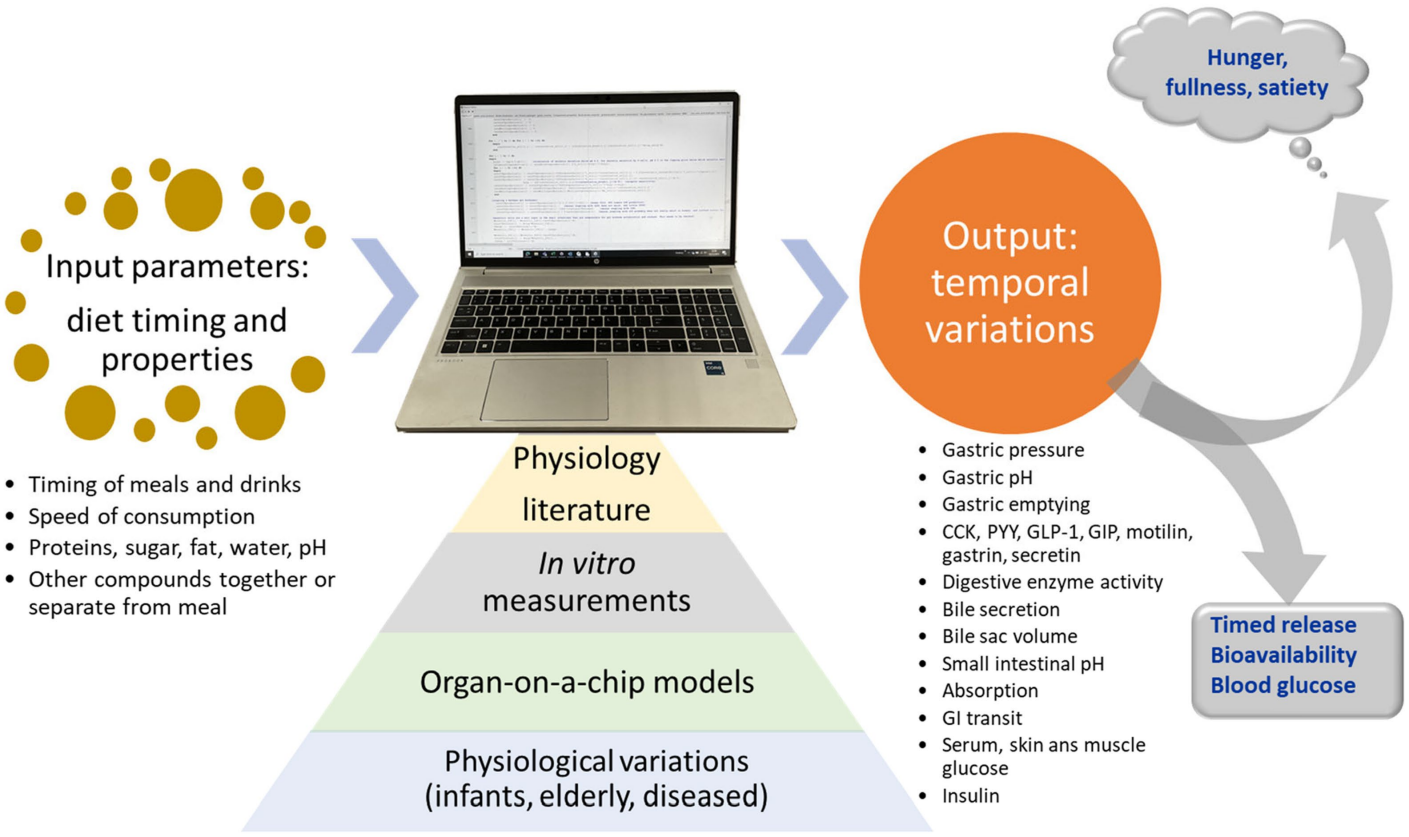
Concept of the mechanistic digestion model (MDM). Based on input from literature, *in vitro* digestion measurements, organs on a chip models, and knowledge about physiologic variations between individuals, the model predicts physiological outputs for meals.

The concept of the current MDM is visualized in Figure 1.

### Properties and behavior of the digestive system

Important for the actual *in vivo* behavior of the alimentary system is how the different digestive organs (such as the mouth, stomach, small intestine, and large intestine) cooperate to break down the food, allowing efficient absorption and how intestinal signals that monitor the progression of digestion, absorption, and the presence of nutrients in the blood optimize the digestive processes (33). Much of this knowledge is available from *in vivo* studies by tracking food intake and swallowing, gastro-intestinal motility, digestive fluid release, and progression in digestion through imaging and sampling techniques. The details of the mechanical breakdown, transport of food material along the alimentary tract, digestion, the process of mixing, and convective and diffusive transport toward the receptor and absorptive cells in the gastrointestinal are of crucial importance for the understanding of how the structure and composition of food materials and pharmaceuticals affect the release and absorption of absorbable nutrients, micronutrients, and pharmaceutical actives. As example, the way solid food materials are fractured into smaller pieces with a larger accessible surface area is a main determinant in the absorption of nutrients from solid foods. Soluble fibers and thickeners increase the viscosity of the intestinal lumen, in this way reducing the mixing with digestive enzymes and the transport of nutrients and actives toward the absorptive epithelia. Anti-nutrients from plant materials can be released and inhibit the digestion and absorption processes of carbohydrates and proteins. The isolation procedures and processing of the proteins into food structures are known to have a high impact on the accessibility of the amino acids of the protein. The efficacy of pre-biotic fibers is highly dependent on their release from the food material and their exposure to the intestinal lumen and to their specific interaction with the wide diversity of intestinal microbial strains. Moreover, the processing of food all along the gastrointestinal tract is regulated by various bio-feedback mechanisms, which may strongly affect absorption and nutritional outcomes.

The digestive system is organized into compartments with distinct functions: mouth, pharynx, esophagus, stomach, small intestine (duodenum, jejunum, and ileum), caecum, colon (ascending, transverse, and descending), and rectum. Some parts are separated by sphincters (throat, gastro-esophageal, pylorus, ligament of Treitz, ileo-cecal, and anus). To most parts digestive fluids are added, sometimes through specific orifices (e.g., sphincter of Oddi through which combined bile and pancreatic secretion enter the lumen of the duodenum). The esophagus, small intestine, and colon have a tubular geometry with properties that vary along the length of the tube.

The gastrointestinal system is lined with muscle tissue that performs tonic contractions and phasic contractions (9). Tonic contractions induce an increase in pressure in the proximal gastric compartment (fundus), produce haustral sacculations that temporarily compartmentalize sections of the large and small intestine, and align with the opening and closure of sphincters. Phasic contraction occurs in the tubular geometries of the esophagus, distal stomach (antrum), and small and large intestines. In the fed state, the phasic contractions result in rhythmic waves that generally progress distally, through neural coupling between excitators of contraction, producing peristaltic waves that mix and grind the luminal content and propel this content distally (10), in this way distributing the nutrients and actives toward the absorptive surfaces of the small intestine and through the colon. The precise progression of peristaltic waves is however complex (6, 8), and state of feeding, individual differences, and various diseases (2), including psychological stress, lead to large differences in these motility patterns (3–5, 7, 12, 34).

## Methods

### Mechanistic modeling of digestion

The MDM presented in this publication has been developed mainly on the basis of existing physiological literature that describes the processes of food intake, gastrointestinal transport, digestive fluid release, food digestion, absorption, nutrient detection, incretin hormone release, and the physiological feedback regulation mechanisms, which include sensations such as fullness and hunger that regulate food intake. A good general introduction to the subject is found in the e-book “The Digestive System” made available by the Colorado State University (35). The parameterization of the model has been performed by using directly measured data for, for example, the kinetic constants of enzyme kinetics or, if these are not available directly, by adjusting the parameters by aligning to the *in vivo* experimental outcomes described in the literature.

The MDM was initially developed on the basis of a literature review by van Aken (37), which describes the main gastrointestinal processes relevant to fat digestion and how they relate to sensations of hunger and satiety. The inherent complexity already involved in the still relatively limited number of processes described in the publication motivated the development of an integrating computer model.

The current MDM is written in Pascal code and describes the sequential compartments: plate or cup, mouth, fundus, corpus, antrum, duodenum before the sphincter of Oddi, duodenum after the sphincter of Oddi, three jejunum compartments, six ileum compartments, and colon. The motivation for this choice is that these compartments represent the various functional units and the number of small intestinal units (duodenum, jejunum, and ileum), roughly representing the relative lengths of these small intestinal regions. The division of the small intestine in a larger number of compartments allows a description of differences along its length in the densities of the various receptor and absorptive cells and the thickness of the mucus border. In addition, the model includes a gallbladder to store bile that is gradually secreted by the liver and released from the gallbladder when activated by neural and hormonal (CCK) mechanisms during feeding, resulting in the contraction of the gallbladder and emptying of bile into the duodenum. Secretin and bile salts stimulate bile salt-independent and bile salt-dependent bile flow, respectively.

For each unit, the amount of an unrestricted number of components is calculated for each compartment. These components include the components ingested from food and formed by digestion (water, proteins, peptides formed by gastric and pancreatic digestion, hydrogen ions, bicarbonate ions, amino acids, mucus protein, and the tracer peptide secreted with pancreatic juice to monitor the presence of digestible proteinaceous materials), tri-, di-, and monoglycerides, phospholipids, fatty acids, various carbohydrates, including glucose, fructose, some rare sugars, starch (including slow and rapidly digestible variants), mucus protein, digestive enzymes, bile salts, and complexes such as the micelles formed from bile salt with long-chain fatty acids. In addition, some tracer variants (such as ^14^C and ^2^H) of food components and their digestion products are defined as separate components. The pH–titration curves of the proteins and other buffering materials are also included. From the concentration of dissolved free hydrogen ions, the pH is calculated for each compartment.

In the current model, all components and compositions are homogeneously distributed over each compartment and the compartments are assumed to be well mixed because this mixing within the compartments is largely driven by the contractile motility of the walls of the alimentary compartments and therefore can be expected to dominate over diffusive transport from the lumen toward the compartment walls. Accordingly, the concentrations are calculated from their masses in each divided by the collective volumes, approximated by isochoric mixing. Because the precise values of the densities are not critical for the modeling results, the densities of the components are estimated or taken from many data sources available in literature. In the current program, the following averaged densities are used: water: 1 g/mL, triglycerides: 0.9 g/mL, glucose: 1.54 g/mL, proteins and peptides: 1.35 g/mL, starches: 1.54 g/mL, fatty acids: 0.9 g/mL, sucrose 1.59 g/mL, pentose sugars: 1.69 g/mL, bile salt LCFA complex: 1.69 g/mL, and citric acid: 1.66 g/mL. For hydrogen ions, a very high density 10^6^ g/mL is chosen because its presence in water is not expected to affect the molar volume of water significantly.

Then, to this main line of luminal units, each small intestinal unit is “lined” with a corresponding epithelial compartment representing the epithelial cells that contain the receptor cells (signaling through the release of gut hormones and exciting simulated nerves) and absorptive capacity. In between the lumen and epithelium is a mucous boundary layer that separates the lumen from the epithelial cells. This stationary mucous boundary represents the “unstirred” layer that water and solutes should pass before they reach the absorptive surface. It should be viewed as a filtering mesh, serving to keep microbes and larger fragments away from the epithelial tissues, but because of this, it also introduces a barrier for the transport/diffusion of the solutes prior to absorption that can be affected by hydrocolloids. For this purpose, for all components a diffusion constant is defined within this mucous layer (37, 38).

Relevant for detection and absorption are the concentrations of the components inside the mucous boundary at the surface of the epithelial layer of the brush border, as calculated from the diffusion constants and the thickness of the mucus layer for each compartment, for which typical values are estimated from literature data (39, 40), and by applying Fick’s law of diffusion. The flow of food components and their digests through the unstirred mucous boundary layer toward the epithelial cells is not purely diffusive, but greatly enhanced by the water flux toward the epithelial cells, averaging to approximately 9 L/day (41). In addition, as described theoretically, the flow toward the absorptive epithelia is also enhanced by the motion of the gut wall that is transferred to additional flow around the villi, through a cyclic process of approximation and separation of groups of villi (42). Both flow processes through the mucous layer strongly increase the absorption rate compared to purely diffusive transport through the mucous layer. If the transport through the epithelial cell layer limits the rate of absorption, this will lead to a concentration polarization of dissolved matter across the mucous layer, tending to increase the concentration close to the surface of the absorptive cells. This, in turn, will increase the rate of enzymatic conversion of nutrients by brush-border enzymes. All modeling of conversions by the brush-border enzymes and nutrient detection and absorption rates are based on the concentrations close to the absorptive surface.

Hydrolysis by dissolved digestive enzymes in the luminal compartments and digestive enzymes bound at the epithelial cells are modeled on the basis of Michaelis–Menten kinetics, which for carbohydrates already includes the effects of inhibitors. The required parameterization is obtained from a large number of *in vitro* studies described in literature [for example, for sucrase, the Michaelis–Menten constant and the inhibition constant for acompetitive inhibition by L-arabinose were derived from the graphs by Seri et al. (43)]. In particular, the current model includes the inhibitory effects of inhibitors for brush-border α-glucosidases (sucrase and maltase) on disaccharide-derived monosaccharide formation (43) and inhibitors of luminal α-amylase that hydrolyze starches (44–46). The model can be extended to include protease inhibitors as well.

The nutrient and hormonal output of the epithelial cell compartments is collected in the common blood compartment of the portal vein, which passes its content through the liver before delivering it to the main bloodstream. In the current model, the removal of hormones from the bloodstream is quantified by a half-time for each hormone in the bloodstream. The literature values reported in the literature vary between studies and between homologs of the various hormones; therefore, although much more detail can be added to the model, reasonable averages were taken for the modeling. Below some relevant references are given for selected hormones.

CCK degradation constant:= 1/3.3 min^−1^ (47); PYY degradation constant:= 1/10 min^−1^ (48); GIP degradation constant:= 1/7.2 min^−1^ (49); GLP-1 degradation constant:= 1/2.3 min^−1^ (49); Ghrelin degradation constant:= 1/10 min^−1^ (50); motilin degradation constant:= 1/15 min^−1^, estimated based on Saito et al. (51); and secretin degradation constant:= 1/(2–3) min^−1^ (52, 53).

Digestive fluids are added to the mouth compartment (saliva), upper stomach compartment (gastric juice and mucus), duodenum compartment (gut wall secretions rich in mucus and bicarbonate) and through the sphincter of Oddi (bile and pancreatic juice), and jejunal and ileal compartments (mucous secretions). The releases of saliva, gastric juice, bile, and pancreatic, bicarbonate, and mucus secretions are calculated by modeling the literature on these secretion rates, including the stimulation and moderation by hormones and nerve signals, in accordance with the literature (54–61). Again, each of these processes is complex and the modeling can be substantially improved. Bicarbonate and mucus secretion by the small intestine are currently described as guess functions, with secretions proportional to serum secretin levels and the deviation from neutral pH at the brush border. The MDM includes the modeling of hormones secreted by enteroendocrine cells in the stomach, pancreas, and small intestine [the so-called gastrointestinal hormones or gut hormones (63)] on stimulation by food components detected by receptors of the endocrine cells and by cross-activation and inhibition by other gastrointestinal hormones. These gastrointestinal hormones control various functions of the digestive organs, partially by acting as neurotransmitters and neuromodulators in the central and peripheral nervous systems (62, 63). Currently included in the MDM are the secretion and degradation of hormones secreted by the gastro-intestinal tract in response to food-derived signals [glucose-dependent insulinotropic polypeptide (GIP) and glucagon-like peptide-1 (GLP-1)], cholecystokinin (CCK), peptide YY (PYY), motilin, secretin, and gastrin. These hormones control alimentary secretions, which includes the release of gastric juice, mucus, and digestive enzymes, and affect intestinal transport (gastric emptying and ileal brake) and also food intake through sensations of hunger, satiety, and appetite. The model has been extended by including a model (65) for blood and skin glucose levels, determined by glucose absorption and conversion of fructose, glucose blood level in the portal vein and main bloodstream, and utilization of glucose by the liver (transformation to glycogen and release from glycogen, regulated by the pancreatic hormone glucagon) and muscle tissues.

### Modeling principles

**Figure 2:**
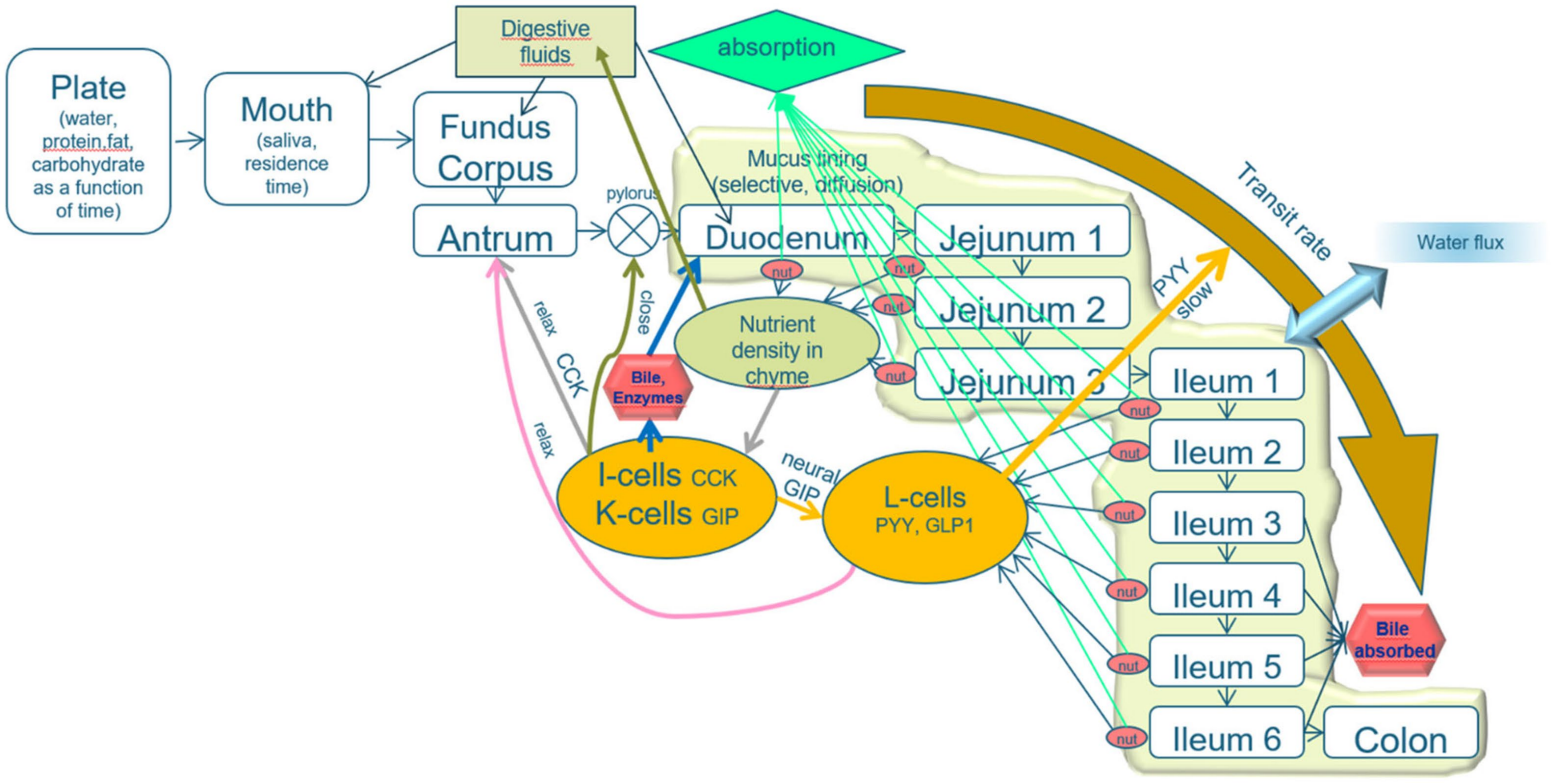
Schematic layout of the mechanistic digestion model. An explanation is given in the text. The model describes the process of alimentation through a line-up of compartments plate, mouth, and three stomach compartments (fundus, corpus, and antrum), the pyloric valve, the duodenum (which is, in fact, subdivided into the parts prior to and after the sphincter of Oddi, from which bile and pancreatic juice are released), three jejunal compartments and six ileal compartments, and a colon. The intestinal compartments are lined with a mucous layer, protecting the epithelial tissues and acting as a sieve that passes only small molecules. A relatively high water flux from the lumen toward the intestinal epithelia increases the transport of small solutes toward the epithelial layer. The epithelial cell layer is equipped with cells that secrete various brush-border enzymes and receptor cells that detect pH, osmotic value, pressure, stresses, and low molecular weight nutrients. The properties of the intestinal compartments (thickness of the mucous layer, number of the various receptor, and adsorptive cells) are varied along the subsequent compartments. The signals from the receptor cells regulate the digestive processes, rates of transit between the compartments, and release of digestive fluids (including saliva, gastric juice, bile, pancreatic juice, and mucous secretion).

The basic layout of the model is that the alimentary system is divided into various compartments with specified functionalities and luminal transport between the compartments. The transformations of the food-derived materials within each compartment and the exchange between compartments are regulated by the output of simulated sensors in all the compartments, based on neural and hormonal signals (Figure 2).

A detailed mathematical description of the complete model is out of the scope of the present publication, only an impression will be given to highlight the basic principles and main compartments. The intention is to give further information in forthcoming publications.

Because almost all variations in the modeled parameters are very slow, leading to only fractional changes in these parameters during typical time intervals of the order of a second, the variations of all parameters can be linearized in time during such time intervals, and the changes of the parameters over such a time interval can be calculated using steady-state equations, for example, using the Michaelis–Menten steady-state limiting equation for enzymatic processes, the steady-state equations resulting from kinetic modeling of transport and absorption phenomena at the brush border, and the steady-state description of the cellular and molecular processes involved in glucose homeostasis.

In the MDM, the stomach is divided into three main compartments, the corpus that receives the food from the esophagus, the fundus that mainly functions as a storage reservoir adapting to the volume of food in the stomach, and the antrum that receives chyme from the corpus and acts as a reservoir for emptying through the pylorus. The three compartments exchange their volumes in a repeating peristaltic motion (three times per minute), during which any soft particular material is ground during the backflow from the antrum to corpus. Gastric secretion, composed of an aqueous solution of 2% mucus protein, 0.5% pepsin (after activation from pepsinogen), and 0.1 M HCl, is added to the corpus compartment at a rate based on measurements by Konturec and Johnson (66):

$\frac{R}{1.5}\;\cdot \;\left(1-\frac{5.5-pH_{\mathrm{antrum}}}{5.5-pH_{\mathrm{antrum},\mathrm{target}}}\right)$ mL/min,

with *pH_antrum_* is the pH of the antrum compartment, *pH_antrum, target_* is the physiologically targeted pH of the antrum compartment, set at 1 in the model, and *R* is a restricting parameter

$$R=1-e^{2-pH_{\mathrm{duodenum}}}\text{,}$$

which has been introduced to protect against excessively low pH in the proximal part of the duodenum, which expresses the effect of secretin signaled by receptors from mainly the first part of the duodenum on low pH.

Gastric emptying is modeled by emptying the antrum into the duodenum by the over-pressure in the gastric compartment and by restricting the size of the pylorus by neural signals and gut hormones (in the current model CCK, PYY, GLP-1, secretin, gastrin, and motilin) released by the intestinal absorptive layers, stimulated by distension, pH, osmotic value, and the detection of nutrients by specific receptors in various compartments of the small intestine. Based on fits to measurements by Tack et al. (67, 68) and Janssen et al. (69),

$$\mathrm{gastric}\;\mathrm{tone}\;\left(\mathrm{mBar}\right)=3+0.0117V_{\mathrm{stomach}}{\left(\frac{CCK}{CCK+1\mathrm{ppm}}\right)}^{4}\text{,}$$

where *V_stomach_* is the total gastric volume (antrum + corpus + fundus) and CCK is the blood serum level of Cholecystokinin expressed in ppm. This gastric tone determines a target antral volume

$$V_{aT}\left(\mathrm{ml}\right)=5+V_{a,\max}\frac{\mathrm{gastric}\;\mathrm{tine}\;}{5\;\mathrm{mBar}+\mathrm{gastric}\;\mathrm{tone}}\text{,}$$

where *V_a, max_* is the maximum antral volume, estimated to be 60 mL.

The antrum empties in the duodenum through the pylorus during the third part of the peristaltic cycle of 1/3 min at a transfer rate

$$J_{\mathrm{antrum}\;to\;\mathrm{duodenum}}\;\left(\mathrm{ml}/\min\right)=V_{\mathrm{antrum}}\left(1-\frac{V_{a}}{V_{\mathrm{at}}}\right)\;\cdot \;F\text{,}$$

where *F* restricts the passage through the pylorus due to a range of stimuli,

$$F=p\;\cdot \;(\sum {\left(EIS\right)}^{-1}\text{,}$$

where *EIS* is an emptying inhibiting stress. Several EISs that will reduce gastric emptying are taken into account, in the current program specifically CCK serum levels, the deviation from isotonicity of the osmotic values of the fluid adjacent to the brush border, a low pH at the proximal duodenal compartment, high GLP-1 concentrations, and viscosity of the fluid in the antrum/gastric tone. The prefactor *p* is a calibration constant derived by fitting the calculated emptying profiles to values measures for a range of fluids with different viscosities and caloric contents by Camps (70).

As reported from a study in mini pigs by Weber and Ehrlein (71) on an isolated part of the jejunum, the jejunal absorption of carbohydrate, protein, fat, and energy demonstrates saturation kinetics, which appears to maintain a maximum rate of energy absorption by the jejunum, in which the jejunum also serves as a temporary storage reservoir using its length. At the same time, the presence of temporarily stored nutrients in the jejunum also reduces the gastric emptying rate, which in this way will avoid an overload in the storing capacity of the jejunum or small intestine as a whole. This effect corresponds to the ileal brake mechanism (72), induced by the detection of nutrients in the distal ileum and proximal colon and signaled by the release of peptide YY (PYY) and glucagon-like peptide 1 (GLP-1). In the MDM, it is assumed that absorption of nutrients by the human small intestine shows similar features. Based on the data points from the pig study, an equation that best describes the physiological results was developed and fitted to the data points, and adjusted to the body weight of humans compared to the mini pigs, as explained by van Aken (35). For each small intestinal compartment, first the absorption rate of each nutrient component *j* in compartment *i* in the absence of other nutrient components is calculated (according to Michaelis–Menten absorption kinetics based on the concentration of the absorbing species at the brush border), after which the value is corrected for the competition in absorption with the other components is corrected by a factor *G*[*i*], which seems to be best represented by the following equations:

$$\begin{array}{c} \mathrm{absorption}.\mathrm{rate}\left[i,j\right]=G\left[i\right]\;\cdot \;\mathrm{absorption}.\;\mathrm{rate}\;\left[i,j\right]\;\\ \mathrm{in}\;\mathrm{the}\;\mathrm{absence}\;\mathrm{of}\;\mathrm{other}\;\mathrm{nutrients}\text{,} \end{array}$$

where

$$G\left[i\right]=\frac{M\left[i\right]}{T\left[i\right]+M\left[i\right]}\text{,}$$

in which *M*[*i*] equals the calculated maximum absorption rate for all nutrient component *i* in kcal/min for compartment, estimated for humans to be approximately 1 kcal per small intestinal compartment with a length of approximately 1 m, and *T*[*i*] is the total target absorption rate in kcal/min for all component in compartment *i* for the case that the absorption of each component would not have been limited by the competition by the other components, hence

$$\begin{array}{c} T\left[i\right]=\sum_{\mathrm{all}\;\mathrm{nutrients}\;j}a\mathrm{bsorption}.\mathrm{rate}\;\left[i,j\right]\;\\ \mathrm{in}\;\mathrm{the}\;\mathrm{absence}\;\mathrm{of}\;\mathrm{other}\;\mathrm{nutrients}\text{.} \end{array}$$

**Figure 3:**
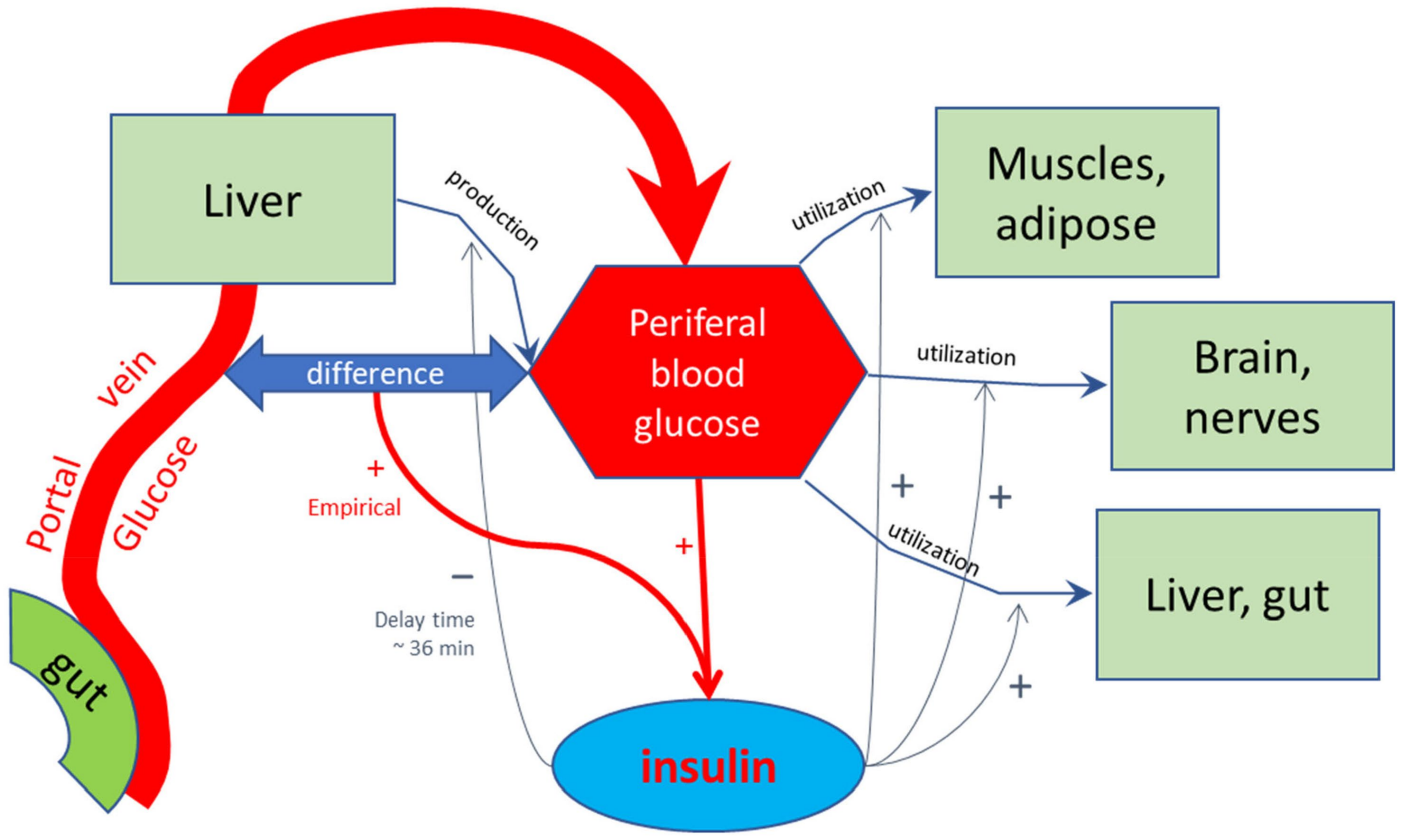
Schematic representation of the model by Toliċ et al. (65) used to calculate glycemic and insulinemic excursions. In this model, glucose is forwarded by the small intestinal epithelial cells into the portal vein, and transported to the liver. The liver feeds the glucose into the peripheral bloodstream, in addition to glucose produced by the liver, among others by hydrolysis of glycogen, which is reduced by increased levels of peripheral insulin with a delay time of approximately 36 min, as found by empirical optimization of the simulated Insulin excursions. Peripheral insulin also stimulates the utilization of peripheral blood glucose by the diverse body tissues. The production and release of regulating hormone insulin into the peripheral bloodstream are stimulated by high glucose levels in the peripheral bloodstream and also hypothetically by a difference between blood glucose levels between portal and peripheral blood. This additional stimulating effect on insulin release may signal a high incoming load of glucose from the gut. As discussed in the main text, such stimulating effect may also be due to the detection of high glucose levels at the mucus border of the absorptive epithelial cells of the small intestine, signaled by gastric inhibitory peptide (GIP), which was not included in the model of Toliċ et al.

For the purpose of calculating the glycemic effects of carbohydrates, the program has been equipped with units that describe the kinetics of passive absorption, transporter proteins across the apical cell membrane of absorptive enterocytes, and the brush-border enzymes maltase, sucrase, α-dextrinase, glucoamylase. Glucose and galactose are co-absorbed with Na^+^ and water by the high-capacity Sodium-dependent GLucose coTransporter 1 (SGLT-1), and Fructose and Glucose by the lower capacity GLUcose Transporter type 5 (GLUT5). Furthermore, in the current MDM, glucose homeostasis is modeled according to Toliċ et al. (65), who described model parameters based on mathematical fits of many *in vivo* and *in vitro* physiological studies, using a model schematically sketched in Figure 3. In this model, the glycemic peak becomes high if the rate of glucose entering the portal vein is high. This fast absorption would lead to a temporary larger difference in glucose concentration between the portal vein and peripheral blood, which would enhance insulin secretion by high peripheral blood glucose concentrations. This model needs to be updated to a more recent model that, among others, includes the enhancing effect on insulin release by GIP, which is released to the portal vein by glucose delivery by the enterocytes to the portal vein as an additional stimulus for insulin secretion (73–76). In this updated model, peaks in serum insulin are related to peaks in the serum GIP concentration.

The overall model, describing the combined effect of all compartments with literature-based set modeling parameters, is adjusted by setting the parameters that describe interactions between the different compartments, optimizing for the agreement with the experimental whole-body responses for adults representing the average adult population. As a result of both optimizations, the resulting model will describe many processes of the alimentary tract in a “one model fits all” fashion, which can also be used to predict the effects of altered physiological states and new food or meal compositions.

Meal data are introduced to the program through a separate text file, produced from a template in the form of an Excel spreadsheet. The spreadsheet allows the definition of a single or multiple subsequent meals, drinks, or snacks at user-defined times and maximum consumption rates (if needed over many days), each meal being defined by a composition of more or less separate compounds that are each defined by their individual components. For each of these compounds, one can define the pH and whether it is a solid or a viscous liquid; in case of a liquid, the viscosity can be defined, in case of a solid its particle size and oral and gastric mechanical breakdown times (mastication and gastric grinding) can be introduced. For each compound, the time of decomposition into its components in the mouth and stomach can be introduced. This allows for large flexibility in the choice of meal compositions in prolonged diets, and the modeling of differences rated to texture, pH, composition, the effect of maximum consumption rate, and appetizers, pre-dishes, deserts, and drinks with the meal. In case of oral medications, in this way also the time of intake with respect to meal timing can be modeled.

The output is again a text file that contains for all components and for each minute the amounts in each compartment and also blood hormone concentrations and estimated sensations of hunger and gastric fullness. This text file can be read by an Excel file that converts the data into a template graph.

## Results

In its current form, the MDM has been used for explaining the physiological effect of the consumption of a variety of foods and has been used to extrapolate and design new experimental studies on the basis of previous results obtained from *in vitro* and *in vivo* studies. In this section, the application of the MDM will be illustrated in two series of simulations. Each simulation delivers an output in an Excel file, containing a multitude of graphs, including temporal variations in compartmental volumes, gastric tone, gastric pH, gastric emptying rate, the contributors inhibiting gastric emptying (antral viscosity, incretin hormones, osmoreceptors, pH, and particle size of solid matter in the stomach), the release of digestive fluids (gastric fluid, intestinal secrete, bile, and pancreatic juice), the concentration of components at the enterocyte border, absorption rates, blood serum glucose and insulin, and serum concentrations of the gastrointestinal hormones. In practice, measured concentrations of the gastrointestinal hormones are highly dependent on the method of analysis, are usually not obtained by continuous monitoring, and appear to vary between individuals, both regarding their baseline values and their increase in fed state. Therefore, only a rough indication of the expected serum values is given. Despite the uncertainty in the precise values of the incretin hormone values, their physiological effects have been coupled in a linear fashion to their modeled physiological effects.

### Simulation 1. Glycemic effects of bread and pasta meals eaten at a variation of consumption rates

Eelderink (77) studied the excursions in serum glucose and insulin for bread and pasta meals with similar macronutrient compositions. The expectation was that the more viscous and dense structure of pasta compared to the structure of bread would lead to a reduced serum glucose excursion. However, somewhat unexpectedly, *in vivo* measurements demonstrated that the blood glucose excursions remained almost similar, but instead, the insulinemic excursion was significantly reduced for the pasta meal compared to the bread meal. To parameterize the differences in structure between the meals, for the modeling, the breakdown of particles of pasta due to mastication and gastric processing was set slightly slower than for the bread meal as the only difference between the meals. As reported previously (33), the model correctly and quantitatively predicts the *in vivo* experimental result, for which the modeling results are shown in Figures 4A,B. We may now use the model retrospectively in order to determine which mechanisms in the model have led to the modeling results. Therefore, Figures 4C–M also show the variation of a selected number of other parameters calculated by the MDM. It should be noted that the interpretation of the modeling results does not give any proof of the cause of the experimental *in vivo* observations, but only gives an explanation of the modeling results, which may need further experimental validation. Nevertheless, the model is based on mechanisms established for studies reported in the literature and may therefore give plausible explanations of the *in vivo* observations.

**Figure 4:**
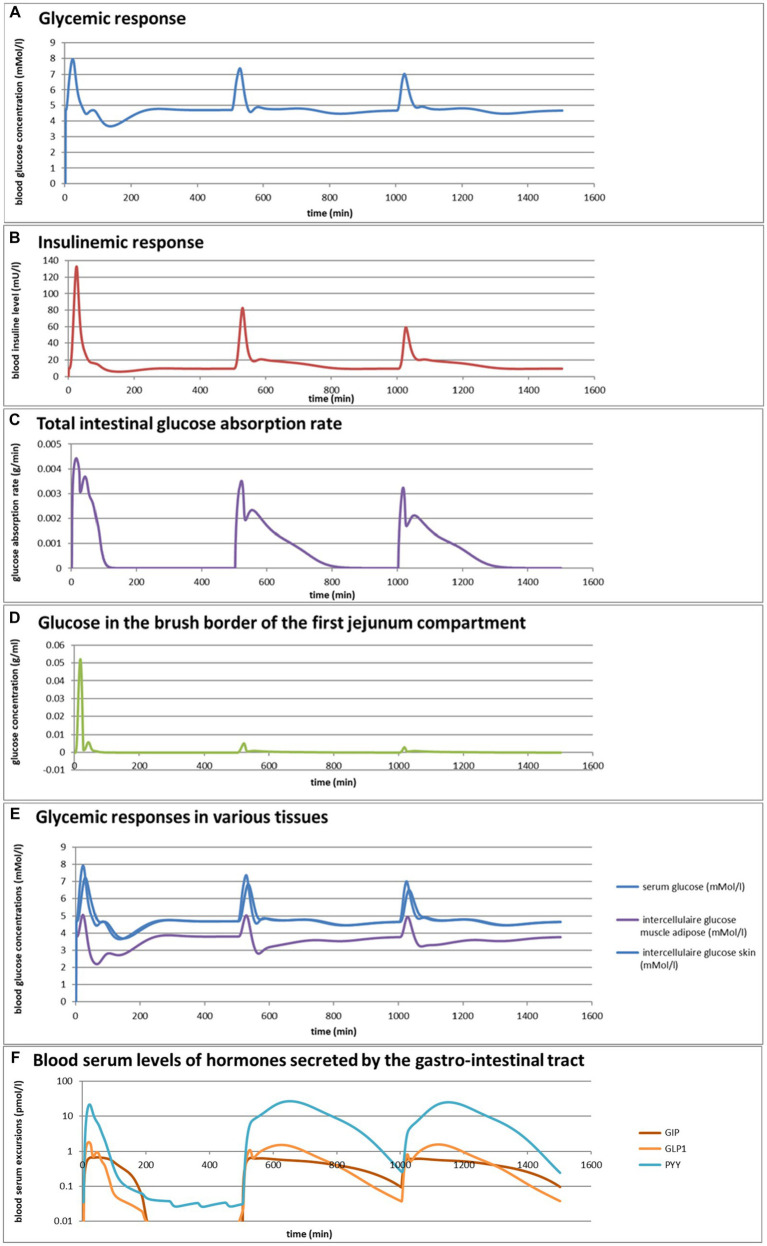


**Figure 4:**
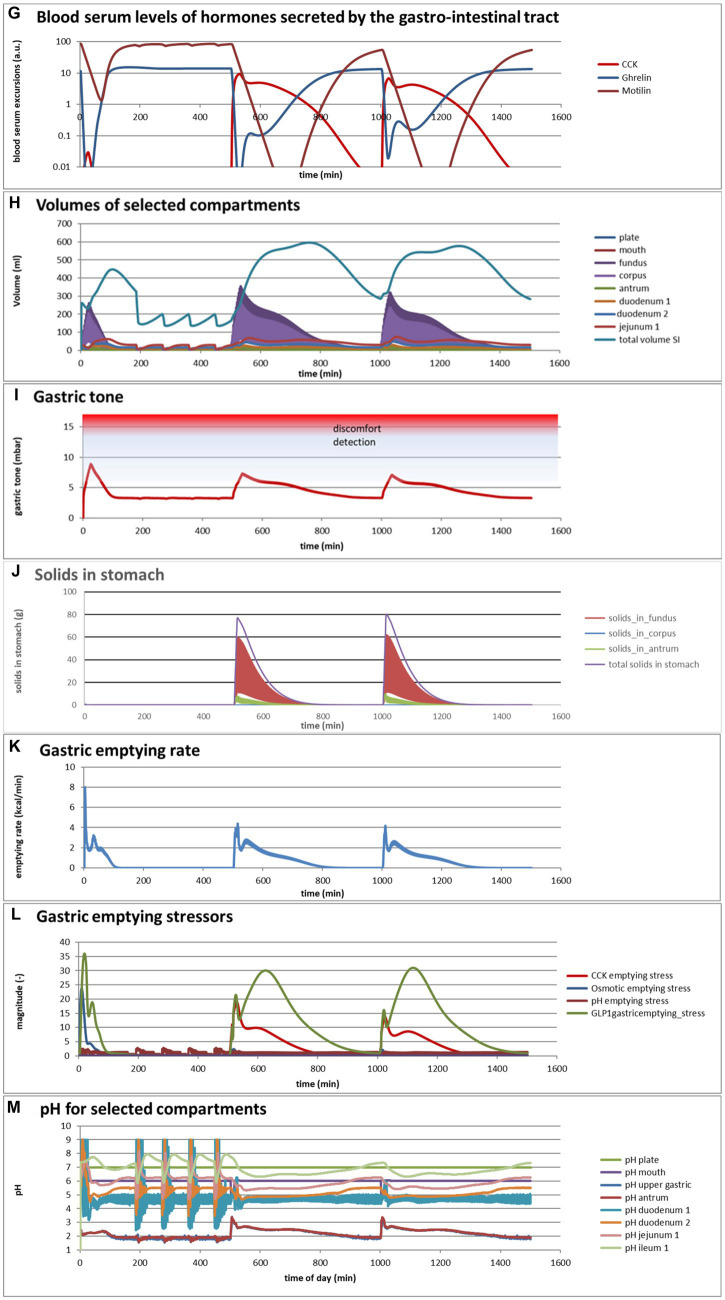
**(A,B)** Excursions of glycemic and insulinemic blood serum values, calculated according to the MDM, **(C–M)** additionally calculated variations in several other parameters (as indicated in the chart titles). Simulated is the effect of three subsequent meals of 50 g of glucose in 250 mL of water (consumption starts at 0 min with a consumption speed of 60 mL/min), a mixed component bread meal of 132 g with 250 mL of water (consumption starts at 500 min, with a consumption rate of 60 mL/min) and a mixed component pasta meal of 119 g (consumption starts at 1000 min, with a consumption rate of 60 mL/min), of which the glucose meals correspond to a glucose tolerance test and the two semi-solid meals correspond to the meals given to a test panel by Eelderink et al. (77). The pH of the three meals has been set to 7.0. The modeled bread meal is composed of 67 g of water, 9 g of fat, and 50 g of rapidly digestible (gelatinized) starch. The modeled pasta meal is composed of 54 g of water, 9 g of fat, and 50 g of rapidly digestible (gelatinized) starch. The red color gradient in **(I)** indicates the expected sensation of gastric discomfort due to an over-pressured stomach, which is thought to relate to feelings of gastric fullness or bloating, in accordance to Janssen et al. (69).

Figures 4A–M show a number of interesting aspects. A clear difference between the glucose meals compared to both semi-solid meals is that the glucose solution initially empties very rapidly from the stomach, followed by a quick peak absorption rate that occurs before the release of insulin that for solid meals moderates the glucose peak. The cause of this is that glucose is swallowed, emptied from the stomach, and absorbed much faster than the glucose produced from starch by amylase hydrolysis. This can be seen from Figure 4C showing that while glucose absorption from the glucose meal has ended completely after approximately 100 min, it is much more sustained after the bread and pasta meals, with the initial peak slightly higher and shorter after the bread meal. Figure 4D shows that only after the glucose meal the glucose concentration near the brush border becomes high, apparently temporarily exceeding the absorptive capacity of the absorptive cells. Figure 4E shows that especially for the sucrose meal a deep and extended dip in the glucose concentration occurs in blood and body tissues, which is especially noticeable in the muscle and adipose tissues; such a dip is not observed in blood and skin tissue after the bread and pasta meals, but nevertheless occurs to some extent in the muscle and adipose tissues. Figure 4F shows that GLP-1 and PYY are elevated for an extended time after the bread and pasta meals, which is because of the presence of fat and protein in both meals, which also reach the nutrient receptors in the ileum and colon and invoke the ileal brake. Figure 4G shows that CCK is only released substantially after the bread and pasta meals, which is related to the presence of fat and protein in these meals, and also that Ghrelin and motilin rise much faster after the glucose meal than after the bread and pasta meals. This is because nutrients have emptied much faster after the glucose meal. Ghrelin will then lead to an upsurge in appetite quickly after the glucose meal, and the high Motilin values will incite the migrating motor complex (MMC), which is a cleaning cycle that in the model increases all transport rates between the compartments and is also known to be felt as a stomach rumble or hunger pangs. Figure 4H shows that the relative volumes of the gastrointestinal compartments much more quickly decrease after the liquid glucose meal than after the semi solid bread and pasta meals, as expected. Remarkably, the volumes decrease slightly faster after the pasta meal compared to the bread meal, which remains to be explained. Figure 4I shows that gastric tone remains acceptably low for all meals, but that the glucose meal is initially felt as more filling (Fullness), but that this Fullness is much more transient than for the other meals. Figure 4J shows that, as expected, solids are only present in the stomach after the bread and pasta meals. Figure 4K shows that, although for all meals gastric emptying rate stabilizes at approximately 2 kcal/min, the presence of solid material in case of the bread and pasta meals reduces the initial rate of caloric emptying, which forms the cause of the initial glycemic peak as shown in Figure 4A. Figure 4L shows that gastric emptying for the glucose meal is mainly restricted by the small intestinal osmotic and GLP-1 responses, while for the two solid meals, it is initially restricted by both the CCK and GLP-1 responses and, after the initial glycemic peak, mainly by the GLP-1 response. Figure 4M shows the calculated variation in pH, showing relatively stable pHs for the different compartments except for the strong variations connected to the MMCs after the glucose meals and rises in the pH of the gastric compartments with the consumption of the more strongly buffering bread and pasta meals, which is due to the presence of protein in these meals.

Comparing the two solid meals, the pasta meals produce a significantly lower insulin peak due to the reduced incretin effect, in which the production of insulin is stimulated by the release of the incretin hormones PYY and GLP-1 during the initial peak in blood glucose, as seen from Figure 4F.

As a shortcoming in the modeling by the current MDM, the effect on both the glucose absorption rate and appearance of GIP is smaller than observed by Eelderink, which is probably due to a too small difference of the oral and gastric structural breakdown rates and because the glucose homeostasis model by Toliċ et al. (65) does not take into account the role of GIP, which needs an update as discussed in the “Modeling principles” section.

### Simulation 2

**Figure 5:**
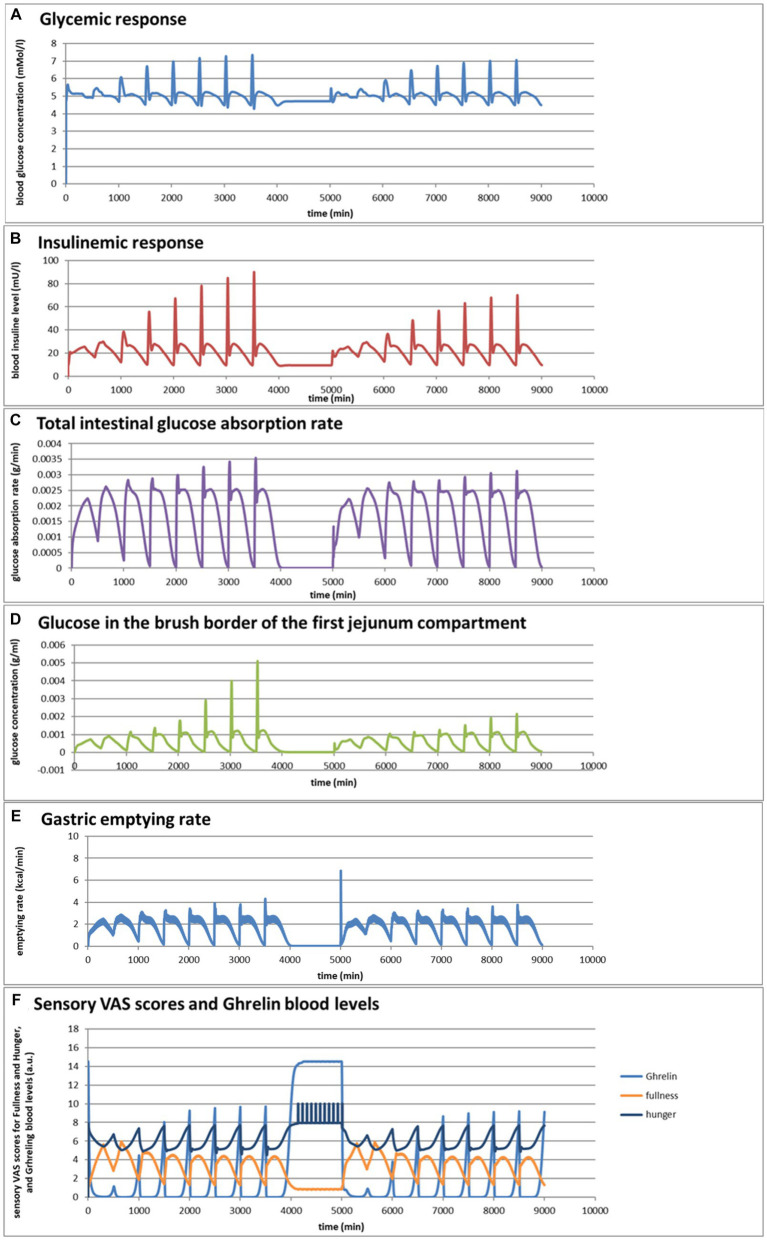
**(A–F)** Selected graphs of simulated outcomes of bread and pasta meals at different consumption rates, modeled for subsequent portions of 300 g of bread meal or pasta meal, with the same composition as in Figure 4. Time of starting points of subsequent meals and consumption rates were for the bread meals: 0 min (1 mL/min); 500 min (2 mL/min); 1,000 min (4 mL/min); 1,500 min (8 mL/min); 2000 min (16 mL/min); 2,500 min (32 mL/min); 3,000 (64 mL/min); 3,500 (128 mL/min); for the pasta meals: 5000 min (1 mL/min); 5,500 min (2 mL/min); 6,000 min (4 mL/min); 6,500 min (8 mL/min); 7,000 min (16 mL/min); 7,500 min (32 mL/min); 8,000 min (64 mL/min); 8,500 min (128 mL/min).

Taking the pasta and bread meals used in the previous example as a starting point, the MDM was used to predict the effects of a variation in consumption rates, which varied broadly for both meals from 1 to 64 mL/min. These predictions, shown in Figures 5A–F, are based on an extrapolation of the successful simulation of the serum glucose and insulin excursions shown in Figures 4A,B, which is possible because the simulations are based on mechanisms. They do not give proof, but instead an indication, of what can be expected as an outcome of an experimental *in vivo* study. For such an *in vivo* study, the MDM results can be used to prepare the experimental conditions (such as the composition of a meal and speed of consumption) such that a measurable significant effect can be expected.

Figures 5A,B show that the larger difference in the insulinemic excursion compared to the glycemic excursion between the two meals found in Figure 4 pertains to all consumption speeds. Figures 5C,D shows that the glucose peak in the total glucose absorption rate and the brush-border glucose concentration only becomes significant at eating rates of 4 mL/min and higher, leading to peaks that are higher after the bread meal compared to the pasta meal, related to a fast initial emptying of glucose from the stomach that is higher for the bread meal compared to the pasta meal. This effect also causes higher values of the incretin hormone levels PYY and GLP-1 during this first peak for the bread meal compared to the pasta meal. Except for the initially peaking glucose gastric emptying rate at higher consumption rates, the feedback control mechanisms lead to a roughly constant gastric emptying rate of approximately 2 kcal/min for both meals at all consumption rates (Figure 5E). Interesting is also the resulting graph for the predicted sensory Visual Analog Scale (VAS) scores for Fullness, which rises less and reaches lower maximum values for increasing consumption speed, whereas after the meal the return of Hunger and the serum Ghrelin concentrations peak at higher values until the next meal (Figure 5F): eating slower would more slowly increase the feeling of Fullness during the meal, but toward a higher value at meal ending, and delay the return of Hunger and high Gherlin levels. The extremes for each meal initially increase with consumption speed but reach a plateau above a consumption speed of approximately 16 mL/min (corresponding to a caloric consumption speed of approximately 35 cal/min). Further increasing the consumption rate does not appreciably increase the maxima in Hunger and serum Ghrelin concentrations, where higher Ghrelin concentrations are thought to be related to increased appetite. (78–81). Moreover, the simulations suggest that at higher consumption rates the pasta meal compared to the bread meal leads to a slightly smaller post-meal sensation of Fullness and lower serum Ghrelin concentrations, suggesting that the pasta meal, at the same consumption rate, will be slightly less filling during and shortly after the meal but also slightly less filling during and shortly after the meal but also slightly delays the return of appetite. In the 1,000-min period separating the sequences between the bread and pasta meals, the simulated subject experiences low fullness, much appetite, and experiences hunger pangs due to the occurrence of migrating motor complex cycles (Figure 5F).

## Discussion

The MTM described in this publication is available as an open-source program currently written in the programming language Pascal, and initiatives are being taken to translate the program to MATLAB and Python. Besides the applications demonstrated in the results section, first attempts have been undertaken to include in the model:

- The way mastication and gastric grinding fracture of solid food materials into smaller pieces with a larger surface area, in this way increasing the rate of digestion and absorption of nutrients from solid food materials.
- The way the viscosity of the gastric and intestinal luminal fluids is increased by thickeners and dietary fibers, and by doing so reduces gastric emptying rate, the mixing with digestive enzymes, and the transport of nutrients and actives toward the absorptive epithelia, resulting in an extended sensation of fullness and a decreased absorption rate.
- The way absorption of amino acids and small peptides, small sugars, and fatty acids from whole meals depends on their formation by digestive enzymes, which are present in the small intestinal brush border, and of which, the secretion is regulated by hormones secreted by the gastrointestinal tract and neural pathways in order to adjust the secretion to need, as signaled by receptor cells in the small intestinal epithelia. These signals also regulate gastrointestinal motility and transport in order to optimize the intestinal residence time for maximum absorption and to avoid overflow into the large intestine.
- The way inhibitors of digestive enzymes decrease nutrient absorption rate and bioavailability. This is of particular importance for food ingredient and food product-producing companies in view of the transition from animal-derived to plant-derived proteins, as many plant feedstock contain these inhibitors as antinutrients purposed as defence against insects.
- The way thickeners, dietary fibers, and inhibitors of digestive enzymes can be used to reduce the fast absorption of glucose from a meal, in this way reducing the glycemic load of a meal, which is of great interest for food-producing companies for the development of foods for diabetic customers.

The intention is to improve, extend, and apply the program by collaboration among a group of experts on various modeling topics. The need for a regular update was described science in the context of the applied models for glucose homeostasis and GIP secretion. The MDM or routines used in the MDM can be used to facilitate more realistic *in vitro* digestion models, for example, for controlling gastric pH, gastric emptying, digestive juice release, and transport phenomena. Regarding *organ-on-a-chip* models, MDM modeling of intestinal fluids can help to define the intestinal fluids reaching the organ models in the fed state and may circumvent the problem of toxicity of experimental intestinal fluids for the receptors and absorptive tissues of organs separated from the gastrointestinal tract. Other potential applications are pre-tests *for in vivo* studies, detailed interpretations of the results of *in vivo* trials, predicting the impact of medical conditions, such as the effect of age-related altered gastric emptying rate, prediction of the potential impact of surgical interventions (for example, altered absorptive surface area by intestinal resurfacing, intestinal or gastric sectioning, and gastric bypass surgery), and the development of improved pharmaceutical and food products with targeted advantages for health.

## Conclusion

The mechanistic *in silico* digestion model (MDM) described here is an attempt to couple complex information and feedback control mechanisms regulating the process of digestion, which gives as output a large number of physiological markers and the environmental conditions in the digestive tract, transport phenomena, absorption, and some post-absorptive processes involved in digestion. The current program is in an early stage of development, and so optimalization of the modeling equations and parameters is needed, but nevertheless already mimics several aspects of *in vivo* digestion. Further development of such a model by collaboration between researchers from various science fields (e.g., mathematics, nutrition, microbiology, human medical science, food and feed sciences, and pharmacology) to share their knowledge and to extend the model with knowledge from *in vivo, in vitro, ex vivo,* and *organ-on-a-chip* experimental studies.

## Data availability statement

The raw data supporting the conclusions of this article will be made available by the author, without undue reservation.

## Author contributions

GA: Writing – original draft.
